# Rapid identification of antimicrobial resistance patterns allows a faster antibiotic adequacy

**DOI:** 10.1186/s13054-017-1797-8

**Published:** 2017-08-04

**Authors:** Adrian Ceccato, Otavio T. Ranzani, Antoni Torres

**Affiliations:** 1 Department of Pneumology, Institut Clinic de Respiratori, Hospital Clinic of Barcelona - Institut d’Investigacions Biomèdiques August Pi i Sunyer (IDIBAPS), University of Barcelona (UB) - SGR 911 - Ciber de Enfermedades Respiratorias (Ciberes, CB06/06/0028), Villarroel 170, 08036 Barcelona, Spain; 2 Seccion Neumologia, Hospital Nacional Prof. Alejandro Posadas, Illia y Marconi s/n (1684), Palomar, Argentina; 3 Pulmonary Division, Heart Institute, Hospital das Clínicas, University of São Paulo, Av. Dr. Arnaldo, 455-Cerqueira César - CEP: 01246903, São Paulo, Brazil

**Keywords:** Antimicrobial resistance, Diagnosis, Intensive care unit

Antimicrobial resistance is an important problem that requires an urgent response from the scientific community [1]. One of the main goals is to develop new molecules and antibiotics able to cover extended and pan-resistant strains. However, strategies are also needed to prevent the development of new resistant strains. The concept of antibiotic stewardship addresses this situation by seeking to increase appropriate antibiotic coverage and to reduce the unnecessary use of antibiotics [2, 3].

Early, appropriate empirical treatment is associated with better survival [4]. Furthermore, if the initial antibiotic treatment is subsequently modified in the light of the culture results, the patient’s outcome does not seem to be affected [5]. Many strategies for improving adequate empirical treatment coverage and for limiting the use of empirical broad-spectrum antibiotics have been developed, such as the use of risk factors or scores to identify patients who are particularly vulnerable to multidrug-resistant germs [6]. The main problem is that these strategies lack external validation and usually have poor predictive performance; they may encourage the overuse of antibiotics or, more importantly, may not improve the appropriate treatment rate [7, 8].

The use of techniques for the rapid etiologic diagnosis of germs and their resistance patterns seems to be the most promising strategy for achieving targeted, fast, appropriate initial treatment and for limiting unnecessary antibiotic use [9]. Various techniques have been developed for this purpose. Polymerase chain reaction (PCR)-based techniques can be performed directly in fresh samples such as respiratory samples (sputum, tracheal aspirate, or nasopharyngeal swab) and blood. These methods can provide valuable information for clinicians aiming to identify a pathogen or looking specifically for a resistant “signature”, or both. They can also establish whether the etiology is fungal or viral. Several other techniques have been tested for fast identification of germs and resistant patterns, such as MALDI-TOF, Finger Print, LAMP, and chromogenic-based methods [10].

In a case-control study, Garnier and coworkers evaluated the usefulness of betaLACTA® in critically ill patients [11]. BetaLACTA® is a specific new chromogenic device for diagnosing third-generation cephalosporin-resistant Gram-negative bacilli (extended-spectrum beta-lactamase-, carbapenemase-, or acquired AmpC-producing *Enterobacteriaceae* species) and must be performed on isolated strains. Controls were patients enrolled prior to the implementation of the technique. The authors found that cases had earlier antibiotic adaptation (43% vs 2%, *p* < 0.01), which shortened the time to escalation in cases of inappropriate empirical treatment (50.5 (48–73) to 27 (24–28) h, *p* < 0.01) and increased antibiotic adequacy (98% vs. 77%, *p* < 0.01). Regarding the resistance detection, only one false-negative was observed. In previous reports, betaLACTA® showed a lower sensitivity for AmpC-overproducing *Enterobacteriaceae* detection than for extended-spectrum beta-lactamases [11].

Among the main advantages of this device are its cost-effectiveness, the fact that the results can be assessed after few minutes, and the fact that no special equipment is required. Nevertheless, Garnier’s study has several limitations, in addition to the study design chosen. The selection of historic controls may have introduced a bias, given the changes in the local flora, in empiric antibiotic use protocols, and in clinicians’ behavior over the years with regard to escalating/de-escalating antibiotics.

The performance of the device has been validated in various studies. One of the main strengths of the study by Garnier et al. was to assess not just the device accuracy but also its impact in clinical practice [11]. Interestingly, the local staff appeared to implement the new technology in their clinical practice relatively fast. It is possible that this “early adopter” profile favored the observation of the clinical impact. Another strength is the fact that the authors searched for a specific resistant mechanism instead of looking for a range of pathogens and resistance patterns. Indeed, this target testing might be more feasible and improve clinicians’ reliability [11].

Other devices for rapid etiologic diagnosis of infection are also available [10]. There are several reports on the use of devices in blood samples in general patients with sepsis, though their performance is only low to moderate and further improvements are necessary [12]. Other clinical studies for rapid diagnosis in community- or hospital-acquired pneumonias have shown better performance than blood samples, mainly because of the inherent differences between blood and respiratory samples [9, 13, 14]. However, one of the main difficulties for clinicians using rapid diagnostic methods in respiratory samples is to differentiate infection from colonization, and to assess the presence of viable bacteria. Other adjunctive methods or biomarkers can help to improve the discriminatory capacity.

Future research should validate this new diagnostic technique through prospective studies and randomized clinical trials. These studies should evaluate not just its accuracy, but also its net benefits and its impact in reducing the use of broad-spectrum antibiotics, the occurrence of adverse events, and the emergence of new resistances, followed by a cost-effective analysis.
